# Precise spatio-temporal control of rapid optogenetic cell ablation with mem-KillerRed in Zebrafish

**DOI:** 10.1038/s41598-017-05028-2

**Published:** 2017-07-11

**Authors:** C. Buckley, M. T. Carvalho, L. K. Young, S. A. Rider, C. McFadden, C. Berlage, R. F. Verdon, J. M. Taylor, J. M. Girkin, J. J. Mullins

**Affiliations:** 1 BHF/University Centre for Cardiovascular Science, University of Edinburgh, Queen’s Medical Research Institute, 47 Little France Crescent, Edinburgh, EH16 4TJ UK; 2 Biophysical Sciences Institute, Department of Physics, Durham University, South Road, Durham, DH1 3LE UK; 3 School of Physics and Astronomy, University of Glasgow, Kelvin Building, Glasgow, G12 8QQ UK

## Abstract

The ability to kill individual or groups of cells *in vivo* is important for studying cellular processes and their physiological function. Cell-specific genetically encoded photosensitizing proteins, such as KillerRed, permit spatiotemporal optogenetic ablation with low-power laser light. We report dramatically improved resolution and speed of cell targeting in the zebrafish kidney through the use of a selective plane illumination microscope (SPIM). Furthermore, through the novel incorporation of a Bessel beam into the SPIM imaging arm, we were able to improve on targeting speed and precision. The low diffraction of the Bessel beam coupled with the ability to tightly focus it through a high NA lens allowed precise, rapid targeting of subsets of cells at anatomical depth in live, developing zebrafish kidneys. We demonstrate that these specific targeting strategies significantly increase the speed of optoablation as well as fish survival.

## Introduction

Classically, the only experimental tools available to achieve targeted cell death were either slow-acting, non-specific pharmacological manipulation of cell types, or the ‘brute force’ killing of cells using either a cryoprobe or direct damage by a focused high-power laser, frequently operating in the ultra violet. Another widely used technique in zebrafish, which uses a joint genetic and pharmacological approach, is the nitroreductase (NTR) system. In the presence of the genetically-encoded bacterial NTR enzyme expressed specifically in cells of interest, the pro-drug metronidazole (MET) is catalytically converted to a cytotoxic DNA cross-linking agent that induces cell death by apoptosis^1, 2^. This protocol has been used to great effect in the zebrafish in systems such as kidney podocytes, the liver and the heart^2, 3^. However, a distinct disadvantage to this approach is the time required for the pro-drug to act, often requiring from 24–72 hrs to induce apoptosis, and a washout of MET can be required to mitigate side-effects^2, 3^.

To remedy this, advances in transgenic techniques now allow the expression of inducible phototoxic proteins in specific cells of interest, such that locally-applied low-power light of the correct wavelength induces apoptosis^4^. The KillerRed protein is one such genetically-encoded photosensitizer where excessive quantities of reactive oxygen species (ROS) are generated in membrane-bound KillerRed-expressing cells upon illumination with 520–590 nm light in the presence of oxygen, causing apoptosis within an hour^5, 6^.

The KillerRed protein induces strong phototoxicity in cells due to the presence of a water channel between the intracellular solvent and the protein’s methylene bridge, facilitating transport of molecular oxygen to the excited chromophore^7^. Generation of radicals within the photosensitizing protein occurs through the interaction between the excited triplet state chromophore electrons and molecular oxygen. It has been shown that for KillerRed, type I energy transfer occurs and that superoxides are the most common cytotoxic agent^8^. A fraction of the generated ROS bleaches the KillerRed protein and the remainder reacts with the cell in which the photosensitizer is expressed, ultimately resulting in oxidative damage to the plasma membrane and leading to cell death. This is a standard indicator of tissue damage in photodynamic therapy, a clinical light-activated procedure that uses localised oxidative damage through photochemical processes to selectively cause cell death^9, 10^.

This optogenetic ablation tool has been successfully employed in a number of different cellular systems. Transient transfection of KillerRed into cultured 293 T human kidney cells followed by illumination for 10 min (535–575 nm excitation filter, 5.8 W/cm^2^) triggered a 40–60% cell death rate with no phototoxic effect observed in DSRed2 controls^5^. When KillerRed was targeted to mitochondria in B16 melanoma cells, almost all cells were killed after illumination for 45 min (535–575 nm excitation filter, 3.3 W/cm^2^)^5^. Mitochondrial-targeted KillerRed-expressing HeLa Kyoto cells were used as photosensitizing agents in a tumour xenograft^11^, wherein irradiation with 593 nm laser light increased tumour apoptosis from 6% to 14%.

Transgenic zebrafish expressing membrane-bound KillerRed have also been developed for *in vivo* studies demonstrating a loss of cell viability correlated with impaired function. Optical illumination of KillerRed expressed in hindbrain rhombomeres 3 & 5 led to significant cell death within the hindbrain, whilst optogenetic ablation of heart cells expressing membrane-bound KillerRed caused reduced pumping efficiency and pericardial edema^4^.

Although this approach has been successfully used *in vivo*, the localisation, activation, quantification and control of this process through light illumination have not been optimised to maximise local cell optoablation whilst maintaining the health of the sample. We therefore developed and characterised a system to generate localised phototoxicity in a tightly-controlled spatiotemporal manner. This is based around a selective plane illumination microscope (SPIM) wherein the combined abilities for single cell targeting and high-speed treatment minimise collateral damage. We also used the inherent perpendicular light arms of the SPIM methodology to introduce a secondary activating beam through the imaging arm. We chose a Bessel intensity profile for this beam to maximise the depth penetration and use the “self healing” qualities of the beam as it penetrates through tissue.

The development and recent advances in the SPIM have revolutionised long-term time-lapse imaging with minimal perturbation to development in larval zebrafish. Good lateral and axial resolution, as well as significantly minimised phototoxic effects, are achieved by optically sectioning the sample using a light-sheet and collecting the resulting fluorescent signal perpendicularly. The light-sheet is typically formed using a cylindrical lens, restricting illumination, and hence fluorescence excitation, to the focal plane of the detection optics. This minimises out-of-focus fluorescence and photo-damage^12^.

Larval zebrafish are ideally suited for investigating fundamental developmental and repair processes *in vivo* since they are optically transparent and can be genetically manipulated with relative ease. The kidney is integral to normal blood pressure control and regulation; co-ordinated physiological systems, notably the renin-angiotensin system, act to maintain homeostasis. At 3 days post fertilisation (dpf) the only cells that express renin are located at the distal point of the anterior mesenteric artery (AMA)^13^ at a tissue depth of approximately 200 μm. These perivascular cells tightly associated with endothelial cells prove a specific, yet anatomically challenging, system in which to demonstrate the ability to precisely control cell death in small subsets of cells with minimum off-target effects. A stable transgenic line with renin cell-specific expression of membrane-tethered KillerRed (*ren*:mem-KillerRed) was therefore established and used to optimise cell-specific *in vivo* optogenetic ablation.

To optimise targeted cell death, we compared the fluorescence loss between three different illumination methods. We used real-time dosimetry as a predictor of light administration efficiency and cellular apoptosis whilst minimising unwanted off-target effects, and confirmed cell death in photobleached KillerRed-expressing cells. The successful integration of a SPIM-independent excitation beam with a Bessel profile into the imaging path of the SPIM enabled unparalleled precision in targeting phototoxicity in a tightly controlled spatiotemporal manner at depth *in vivo*. We demonstrate that these specific targeting strategies significantly increase the speed of optoablation whilst simultaneously increasing the fish survival rate. This has not previously been possible for anatomically deep tissues such as the kidney, and provides new opportunities for biomedical research including developmental biology and pathophysiology.

## Results

**Figure 1:**
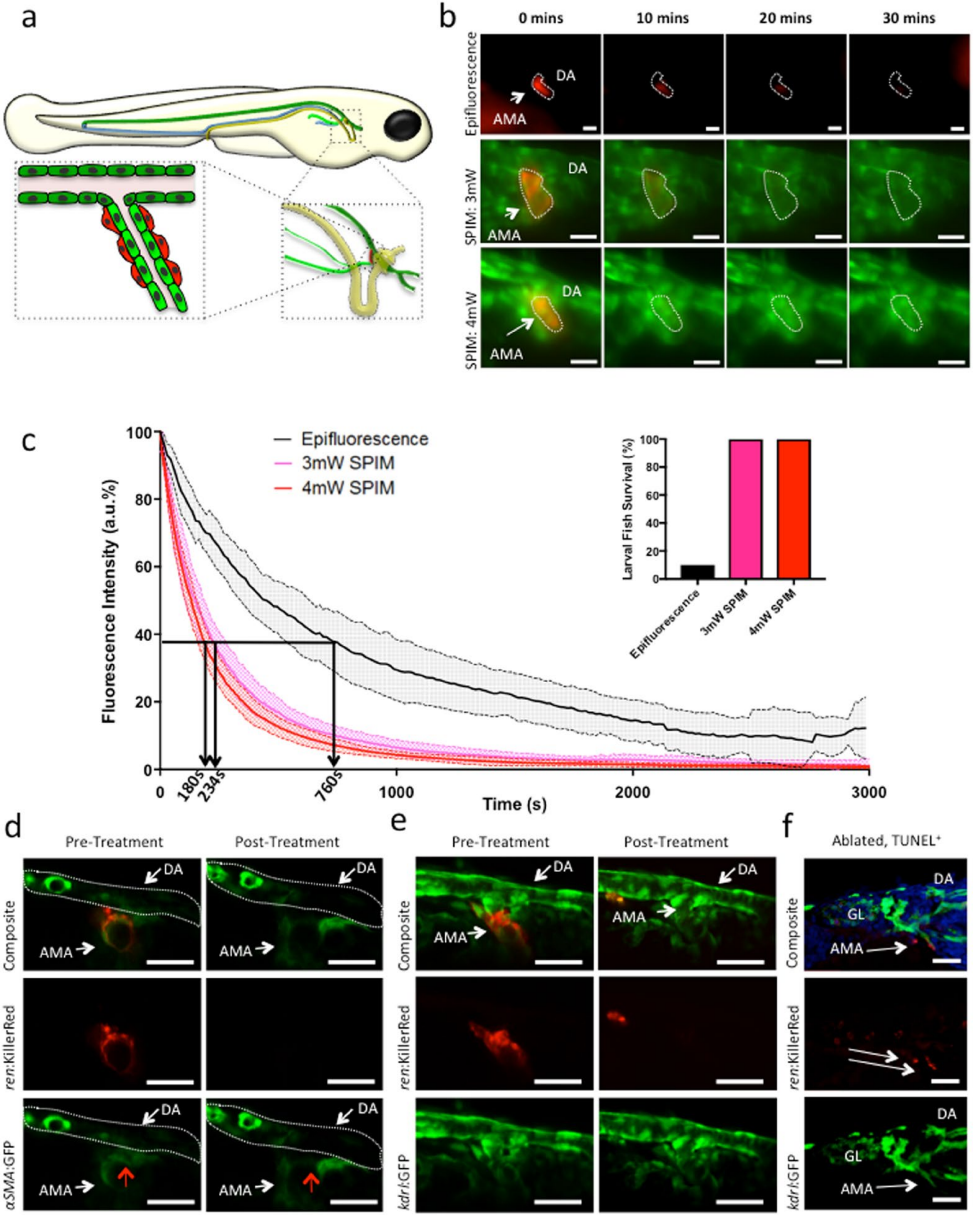
Evaluation of KillerRed optical targeting *in vivo* using epifluorescence and SPIM. (**a**) Schematic showing perivascular renin cell location in 3 dpf zebrafish. Mural renin cells are shown in red, arterial vessels in green, veins in blue, and the glomerulus (GL), nephron and cloaca in yellow. The dorsal aorta (DA) is distinguished from the anterior mesenteric artery (AMA) by using dark and light green to represent them, respectively. (**b**) *ren:*mem-KillerRed cells along the AMA budding off the DA in 3 dpf *ren:*mem-KillerRed;*kdrl*:GFP fish were illuminated using either an epifluorescence microscope (top panel, mCherry filter, 1hr), or the SPIM light-sheet at 3 mW or 4 mW (bottom panels, maximum intensity projections, 561 nm laser, 1hr). Representative images from 0, 10, 20 and 30 min illumination are shown. (**c**) Graphs showing the fluorescence intensity as a percentage of maximum intensity (arbitrary fluorescence, a.u.), plotted against time and averaged over n = 6 fish (with 95% confidence intervals, indicated by the filled dashed lines). Arrows and rotated times at foot of arrows indicate the 1/e value. Inset: percentage of fish surviving following illumination by epifluorescence or light-sheet microscopy for 1 hr (n = 18). (**d**) Morphological changes of *ren:*mem-KillerRed cells were assessed using multiphoton microscopy (MPM). MPM images of 3 dpf *ren*:mem-KillerRed;*αSMA*:GFP renin-expressing cells were taken prior to- and post-treatment using the SPIM light-sheet. Images are presented as single depth slices. Red arrows indicate renin cells. (**e**) Endothelial cell structure prior to- and post-light treatment using the SPIM light-sheet was assessed using MPM in 3 dpf *ren*:mem-KillerRed;*kdrl*:GFP fish. Images are presented as maximum intensity projections. (**f**) Apoptosis of *ren:*mem-KillerRed cells was confirmed using an Apop-Tag *in-situ* TUNEL staining kit on 15 μm sections of 3 dpf *ren:*mem-KillerRed;*kdrl*:GFP larvae illuminated with the SPIM and fixed immediately. White arrows designate apoptosis-positive post-treatment KillerRed^+^ cells. Images show a maximum intensity projection over 5 μm using confocal light scanning microscopy. All scale bars represent 30 μm.

**Figure 2:**
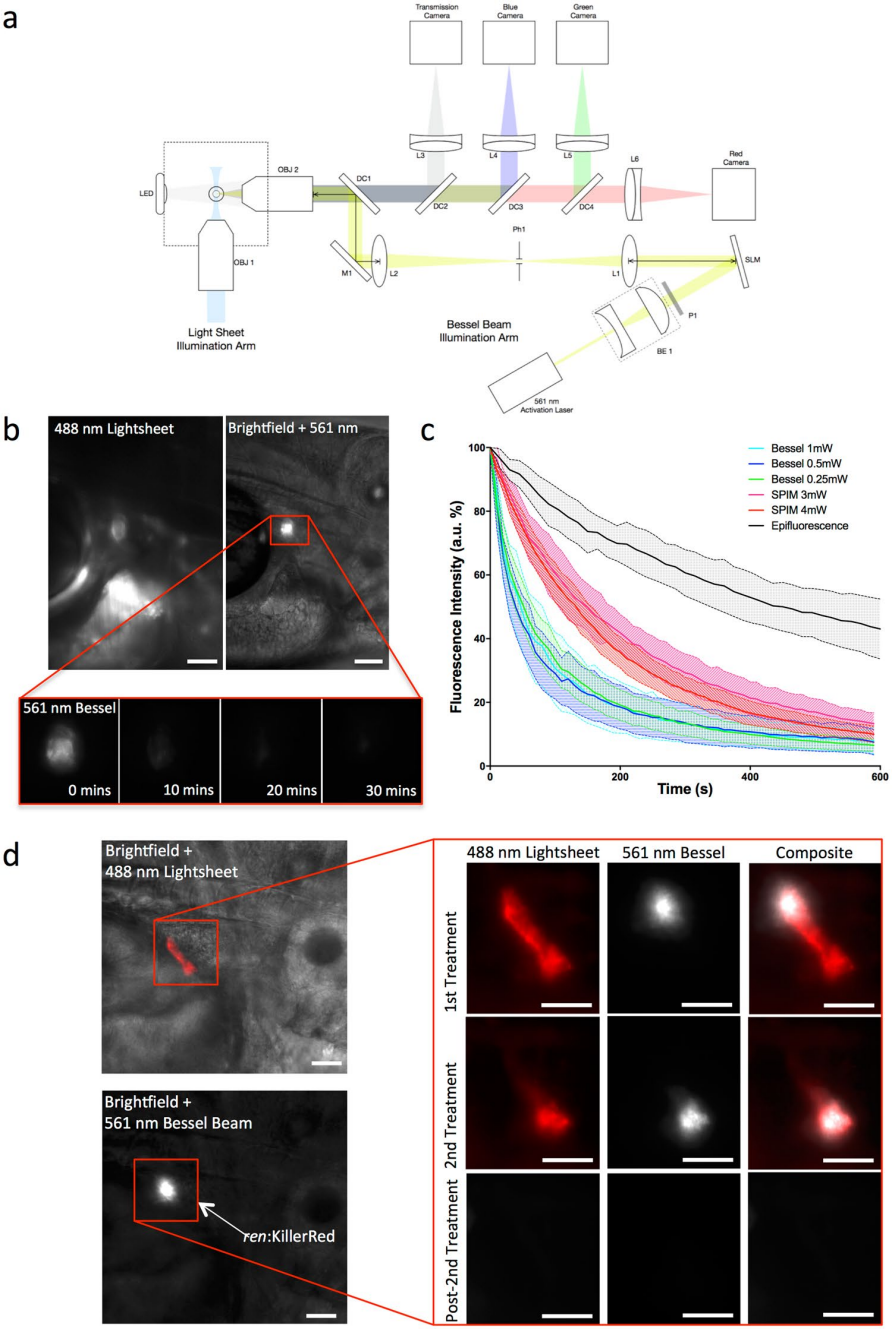
Introduction of a Bessel beam into the imaging path of the SPIM set up allows rapid, localised targeting of KillerRed-expressing cells *in vivo*. (**a**) Schematic diagram of the imaging path of the SPIM system, for fluorescence imaging, with the integrated Bessel beam for cell targeting. OBJ1, OBJ2 are objective lenses; LED, light emitting diode; DC1, DC2, DC3, DC4, dichroic mirrors; L1, L2, L3, L4, L5, L6, lenses; SLM, spatial light modulator; BE1, beam expander; P1, polarizer; Ph1, pin hole; M1, mirror. Coloured lines shown in light blue, blue, green, red, grey and yellow represent light paths for the incident SPIM light-sheet, excited blue fluorescence, excited green fluorescence, excited red fluorescence, imaged transmission light and incident Bessel beam illumination, respectively. (**b**) KillerRed cells in 3 dpf *ren*:mem-KillerRed larvae (indicated by the red box) were located using low power, low frame-rate 488 nm light-sheet excitation (top left panel). The 561 nm Bessel beam was translated to the correct position and superimposed with brightfield illumination for a single frame (top right panel). Brightfield illumination was then turned off and a fluorescence emission image acquired every 10 s using the Bessel illumination until the fluorescence intensity had decreased to the background level approximately 10 min later (representative images shown in bottom panel). (**c**) Graphs showing the fluorescence intensity as a percentage of the maximum intensity (arbitrary fluorescence units, a.u.), plotted against time and averaged (with 95% confidence intervals, indicated by the filled dashed lines). Cells were targeted using the Bessel beam with incident powers of 1 mW, 0.5 mW or 0.25 mW (as measured at the back aperture of OBJ2). The fluorescence intensity measured from acquired images and represented as a percentage of the maximum value, averaged over between n = 10 and n = 12 fish. This is represented individually for each power, and as a comparison between the three different targeting methods: epifluorescence illumination, SPIM light sheet illumination and Bessel beam illumination. Data has been truncated at 600 s to clearly show the initial rapid decay when using Bessel illumination. (**d**) SPIM image showing subsets of KillerRed-expressing cells, which were targeted using the Bessel beam. A representative example is given where the correct location of cells was confirmed using the 488 nm light-sheet, and the 561 nm Bessel beam translated to the dorsal end of the AMA. Relative location within the fish is demonstrated by co-localising fluorescence signal with a brightfield image. Light treatment was performed, targeting a subset of cells (shown before 1^st^ Treatment in the top panel) along the AMA and the decrease in fluorescence intensity confirmed using the light-sheet (shown before 2^nd^ Treatment in the second panel). The remaining cells, which showed no decrease in fluorescence intensity after the first treatment, were then targeted for a second treatment. No evidence of KillerRed expression remained after the second light treatment (shown in the bottom Post-2^nd^ Treatment panel). All scale bars represent 30 μm.

To evaluate the optical targeting efficiency of the SPIM light-sheet, KillerRed^+^ kidney cells in *ren*:mem-KillerRed;*kdrl*:GFP fish (schematic, Fig. 1a) were illuminated using either standard epifluorescence microscopy or our bespoke SPIM system (see Fig. 2a). For epifluorescence experiments, we used a mCherry filter set that was well-matched to the excitation spectrum of KillerRed. A light power of 35 mW was measured at the focal plane, corresponding to an approximate intensity of 4.5 W/cm^2^, which was applied to the same region of the fish over the entire light treatment.

The decrease in KillerRed fluorescence during illumination with epifluorescent light was measured from images collected over time (Fig. 1b, top panel), and compared to that of the targeted illumination performed using the SPIM light-sheet (λ = 561 nm) at 3 mW and 4 mW (Fig. 1b, bottom panels). This corresponds to an approximate intensity of 0.37 kW/cm^2^ and 0.5 kW/cm^2^ for the light-sheet used at 3 mW and 4 mW, respectively, though the plane was continually scanning across the volume of interest. The improvement in image quality when using the SPIM was evident, allowing imaging in two spectrally separate channels with good lateral and axial resolution. The cell boundaries could clearly be resolved and surrounding cells observed during photobleaching (Supplementary video 1). The decrease in fluorescence intensity was quantified and plotted using 95% confidence intervals error bars (Fig. 1c). Photobleaching efficiency - the time for fluorescence emission to fall to 1/e of its initial value - improved significantly using light-sheet compared with epifluorescence illumination. On average, epifluorescence photobleaching fell to 1/e at 760 +/− 250 s compared to the 234 +/− 50 s and 188 +/− 40 s taken to reach this value when using 3 mW and 4 mW SPIM illumination, respectively. No significant power dependence on the rate of the light-sheet photobleaching effect was observed over the power ranges used. Importantly, the survival rate for fish illuminated using the light-sheet was 100% compared to only 10% (n = 18) of fish surviving the epifluorescence illumination protocol (Fig. 1c, inset).

To confirm that the reduction in fluorescence intensity was linked to cell death, cellular morphology was assessed using multiphoton microscopy (Fig. 1d). Multiphoton images of KillerRed^+^ renin cells were acquired in 3 dpf *ren*:mem-KillerRed;*αSMA*:GFP fish before phototoxicity-inducing SPIM illumination (Fig. 1d, PreAblation panels). The fish were transferred to the SPIM for KillerRed activation and cell optogenetic ablation, followed by 1 hr incubation at 28 °C before multiphoton images were retaken (Fig. 1d, Post-Treatment panels). Alterations in cellular morphology and a decrease in *αSMA*:GFP signal were evident (red arrow), and the *ren*:mem-KillerRed signal was eliminated. Perivascular renin cells are tightly associated with the underlying endothelium, therefore a similar experiment was performed on 3 dpf *ren*:mem-KillerRed;*kdrl*:GFP fish to assess endothelial cell viability post-light treatment (Fig. 1e). Endothelial cells and the AMA remained viable and patent post-illumination, confirming the specificity of the light-induced damage to the renin-expressing cells. Apoptotic cell death within renin cells along the AMA of 3 dpf *ren*:mem-KillerRed;*kdrl*:GFP fish after 1 hr of light treatment was confirmed using an anti-digoxigenin (anti-dig)-based Apop-tag fluorescence TUNEL staining (Fig. 1f). Anti-dig-rhodamine^+^ cells were present along the AMA (indicated with arrows), with little off-target staining present. Additional evidence is provided in Supplementary Figure 1; acridine orange is an intravital dye that stains all cells, but accumulates within early apoptotic cells to stain the nuclei bright green due to chromatin condensation^14^. At the end of light-sheet treatment, bright signal accumulation can be seen within cells that were KillerRed^+^ prior to treatment (Supplementary Figure 1a). Furthermore, whole mount anti-Dig^+^ staining visualised with Nitro Blue Tetrazolium showed appropriate signal in the correct location (Supplementary Figure 1b).

To improve the targeting of KillerRed^+^ cells *in vivo*, a Bessel beam was integrated into the imaging light path of the SPIM (Fig. 2a). In this configuration, a separate 488 nm laser was available to form the light-sheet, which was used at low power to locate KillerRed^+^ cells without photobleaching and enabled accurate targeting of the 561 nm Bessel beam to the region of interest (Fig. 2b). Images were acquired every 10 s until no KillerRed^+^ fluorescence was detected (Fig. 2b). The decrease in fluorescence intensity was measured over time following illumination with the Bessel beam at 0.25 mW, 0.5 mW and 1 mW (Fig. 2c), corresponding to approximately 1.5 kW/cm^2^, 3 kW/cm^2^ and 6 kW/cm^2^ intensity at the sample, respectively. Plotting this against the decay curves of the epifluorescence- and SPIM-illuminated embryos shows that using the Bessel beam allows rapid and reproducible targeting between fish. Photobleaching efficiency showed no significant difference between incident laser powers; the time taken to reach the 1/e value was 81 s, 65 s and 88 s for the 1 mW, 0.5 mW and 0.25 mW incident laser powers of the Bessel beam, respectively (Fig. 2c). As with illumination using the light-sheet, the fluorescence intensity change was not dependent on laser power, with the fluorescence decrease curves falling within one standard error of each other.

Targeting subsets of KillerRed^+^ cells along the AMA was successfully accomplished for the first time (Fig. 2d), allowing targeted cell optogenetic ablation within vessels of approximately 50 µm in length. When the Bessel beam was situated at the distal end of the AMA segment containing KillerRed^+^ cells (Fig. 2d, 1^st^ Treatment panel), a small area was photobleached in under 10 min. Comparison with the remaining fluorescence, targeted in the second treatment (Fig. 2d, 2^nd^ Treatment panel), strongly suggests that a single cell was targeted in the first treatment since no bleaching of the lower end of the AMA occurred. Again, total bleaching of the second region was performed in fewer than 10 min. The bleaching of all the fluorescence signal was confirmed after completion of the illumination protocol (Fig. 2, Post-2^nd^ Treatment panel).

**Figure 3:**
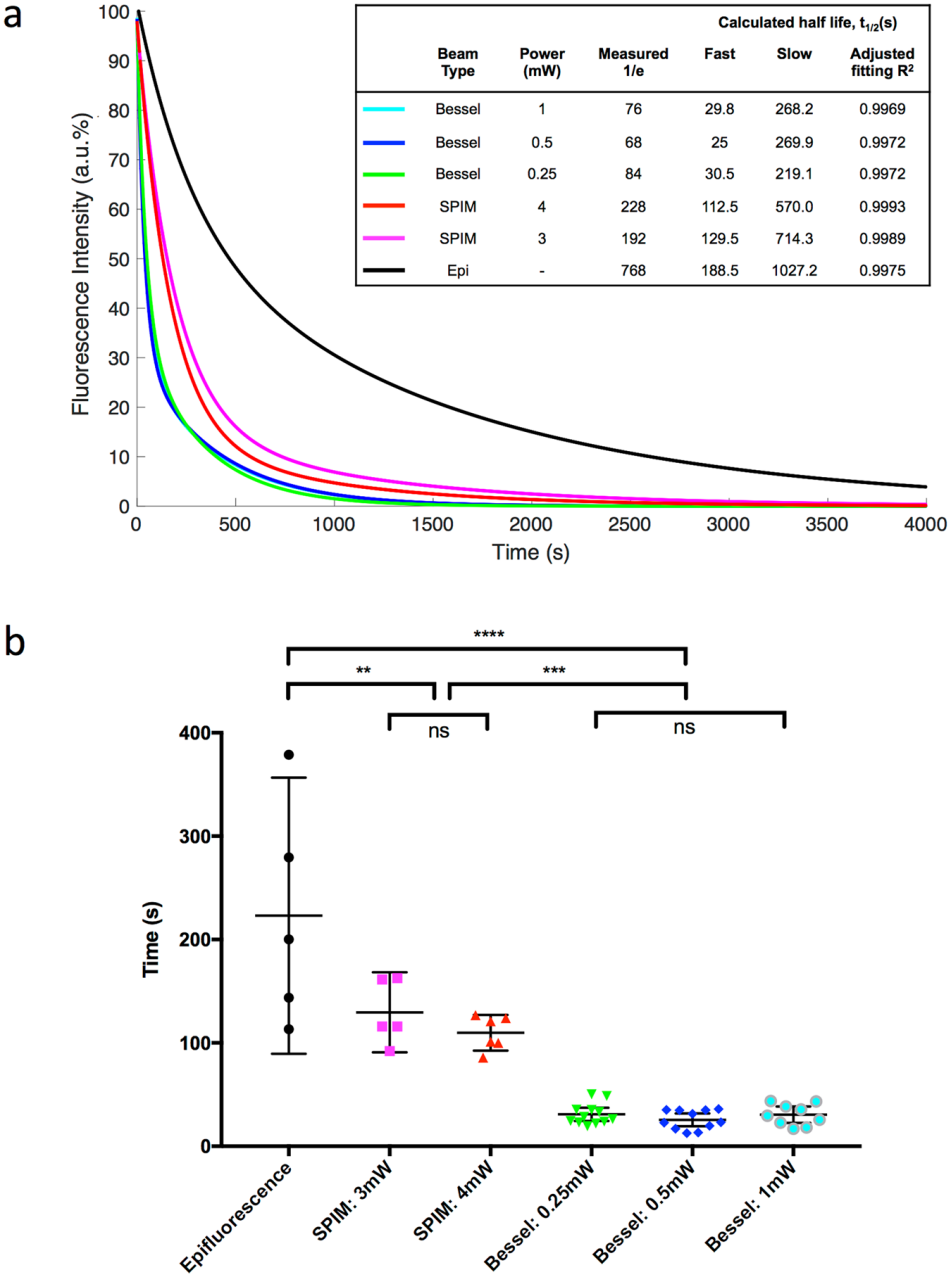
Fitting of decay curves highlights distinction between targeting speed. (**a**) Averaged fluorescence intensity profiles for each group were fitted to two-term exponential functions. The calculated fast and slow decay curve half-lives were calculated and are show, along with the measured time for the curve to reach 1/e. The curve fitting precision is represented by the adjusted R2 value. (**b**) Individual fluorescence intensity decay curves were fitted to two-term exponential functions for each sample. KillerRed photobleaching follows this two-term decay curve extremely well, comprising a fast and a slow decay component. The half-life was calculated from the fast term of each fitted curve, and averaged for each group. Individual points are plotted, with error bar designating the 95% confidence intervals. **p < 0.01, ****p < 0.0001.

Fitting the averaged decay curves to exponential functions showed the different targeting characteristics of the three illumination methods (Fig. 3a); epifluorescence was slow and non-specific, SPIM light-sheet illumination was faster and allowed a volume of cells to be targeted and the Bessel beam targeted subsets of cells extremely rapidly. To quantify this, each curve was fitted with a two-term exponential, and the half-life of the fast term calculated, averaged for each group and compared between groups (Fig. 3b). Epifluorescence illumination provided the slowest decay, shown by the longest half-life of the three methods, 223 s on average. It also showed the widest spread, indicating the lack of reproducibility of the targeting. SPIM light-sheet illumination tightened the reproducibility and significantly decreased the half-life of the decay terms to 130 s for 3 mW illumination and 110 s for 4 mW (p = 0.0023), the different powers of which were not statistically different. However, the Bessel beam illumination was by far the most reproducible and rapid targeting method. The average half-life of the fast term was 24 s for 0.25 mW illumination, 26 s for 0.5 mW illumination and 31 s for 1 mW illumination, which were not significantly different from each other. This was significantly faster than the light-sheet illumination (p = 0.0002) and epifluorescence illumination (p < 0.0001), and was highly reproducible.

## Discussion

The application of our novel SPIM system allows the rapid generation of phototoxicity in a tightly controlled spatiotemporal manner. Through the use of the genetically-encoded photosensitizer fluorescent protein KillerRed, this phototoxicity response can be manipulated to cause targeted cell death. By incorporating a Bessel beam, we are able to induce rapid and highly-localised cell death, coupled with time-lapse imaging capabilities separating imaging illumination from cell targeting.

Using our innovative set up, we demonstrate two independent methodologies for targeted cell death with minimal off-target damage to the sample. The first involved continuous scanning of the light-sheet over the volume of KillerRed^+^ cells for 60 min, providing excellent imaging quality and axial information whilst inducing targeted cell death. This method has the advantage that it can be achieved on a standard SPIM microscope without any modification to the optics. Whilst the power required to illuminate the deep-set renin cells using epifluorescence microscopy was such that the fish did not survive the illumination protocol, the use of the SPIM allowed specific and sensitive targeting. This was particularly highlighted by the fact that the underlying endothelium remained intact. Renin cells are perivascular-like and are intimately linked with the underlying endothelium through peg and socket junctions; if any off-target effects were to be seen, it would be expected to be in such closely associated cells. Evidence from Fig. 1e and Supplementary video 1 show that this is not the case, indicating that the targeting is restricted to the KillerRed-expressing cells.

The second method involved the application of a Bessel beam, in combination with a light-sheet, to locate regions of interest in order to target individual subsets of cells. A Bessel beam is a ‘self-healing’, non-diffracting focal line of light with a central maximum that is resistant to diffractive spreading^15^. These distinctive properties of Bessel beams have been exploited with great success; for example, the long focal depth over large axial distances has been used for cellular photoporation^16^, and the beam has been scanned to create light sheets sufficiently thin to achieve isotropic 3D resolution^17^.

The use of the Bessel beam not only improved the ability to target much more precisely than with the light-sheet, but also increased the speed of targeting by approximately an order of magnitude when compared to epfiluorescence illumination. By integrating the Bessel beam through the imaging arm of the SPIM and decoupling it from the light-sheet illumination, we were able to use a high NA objective (0.8, compared to the 0.3 NA of the illumination objective) to launch the Bessel beam, producing a highly localised targeting beam that is more precise and a set up that is more flexible.

In 3 dpf larvae, renin is localised to the AMA, which branches off the dorsal aorta^18^. The distal end of the AMA is approximately 200 μm from the dorsal surface of the larvae, and is therefore extremely difficult to image and target. The successful targeting of subsets of renin cells along the AMA demonstrates the power of using the Bessel beam in conjunction with genetically-encoded photosensitizing proteins. This is particularly apparent when comparing the targeted illumination of the SPIM and Bessel beam with ‘bulk’ epifluorescence illumination, where the untargeted high-power illumination required to illuminate the deep-seated renin cells led to a low survival rate. Using our powerful, targeted cell-death technique, we will be able to explore the functional roles of renin cells with respect to the surrounding vasculature, blood pressure and kidney physiology. Our accurate targeting and quantification methodology leading to localised damage and high fish survival rates indicates that the method is suitable for other organs and cell types within the developing embryo as appropriate transgenic animals become available.

Taking the results from the curve fitting together with the qualitative information gained, our results show that both methods are more effective at photobleaching, and less harmful to the fish, than epifluorescence illumination. It is interesting that all curves fitted more accurately to two-term rather than single-term exponential functions. There is evidence to suggest that the two-term exponential decay of photosensitizing proteins may correspond to distinctive contributions of the oxygen concentration variation during light administration^9^, which could be used to probe the oxygen dynamics during photodynamic activity.

The cellular localisation of KillerRed expression is important for effective phototoxicity generation. Mitochondrial-, histone H2A- and membrane-bound KillerRed, as in the present study, have been shown to effectively mediate rapid cell death, possibly due to direct oxidation of membrane lipids^5, 6, 19^. Conversely, cytosol-bound KillerRed produces a weak phototoxic effect^5^.

Implicit dosimetry was used throughout the study, since the decrease in fluorescence intensity acts as a proxy measurement for ROS generation and hence cellular apoptotic response. Although a dependence on power was expected for the photobleaching characteristics, this was not seen when illuminating with the Bessel beam. A slight but non-significant decrease in the 1/e value was seen when illuminating with a 3 mW compared to 4 mW beam. This would suggest that we might be saturating the photosynthesizing photobleaching reaction, with either the local oxygen or photosensitizing protein acting as the limiting factor.

In conclusion, we have developed a method to generate phototoxicity *in vivo* in a rapid and precisely controlled spatiotemporal manner with minimal off-target perturbation to closely-associated endothelial cells. These advances will transform the landscape of targeted cell ablation, enabling the study of discrete cells (e.g. renin progenitor cells, pericytes and immune cells) in the development and pathophysiology of previously inaccessible tissues with the absence of any detectable off-target damage. The ability to modulate the level of induced damage in a dose-dependent manner within minutes has wide-ranging applications across models used within biomedical research, particularly when coupled with long term, low perturbation imaging capabilities.

## Methods

### Fish lines and husbandry

Experiments were approved by the University of Edinburgh Animal Welfare and Ethical Review body (AWERB) and conducted in accordance with the UK Home Office Animals (Scientific Procedures) Act 1986. Fish (*Danio rerio*) were maintained at 28.5 °C, as previously described^20^. Embryos were staged according to Kimmel *et al*.^21^ and anesthetised with 40 µg/ml tricane methanesulfonate. The established *αSMA*:GFP^22^ and *kdrl*:GFP^23^ transgenic lines were used to label smooth muscle cells and endothelial cells, respectively.

### Generation of Tg(ren:mem-KillerRed) fish

Tg(*ren*:mem-KillerRed) was created using Three-way Gateway cloning (Invitrogen) and the Tol2kit^24^. Membrane bound KillerRed (Evrogen, Cat.# FP966) was driven by a previously characterised 6.46-kb DNA sequence^13^ immediately upstream of the *ren* translational initiation site^18^. An entry clone containing the *ren* promoter sequence was recombined with mem-KillerRed and a simian virus 40 polyA into the Tol2kit destination vector pDestTol2CG2 (containing tol2 ends and cardiac myosin light chain:GFP). Plasmid DNA was co-injected with transposase mRNA and a single founder fish was used to establish a stable line. The expression of *ren*:mem-KillerRed was comparable to that of *ren*:LifeAct-RFP^13^.

### Epifluorescence Microscopy

A Leica MZ16 F stereomicroscope with top lighting was used. The light source was a 100 W high-intensity mercury burner lamp. Standard mCherry (578 nm/46 nm) and RFP (549 nm/18 nm) filters were used.

Epifluorescence optoablation process: *ren:*mem-KillerRed larvae were oriented laterally in a petri dish on their left hand side to provide the best imaging angle for renin, in 0.5% agar covered with system water. The mCherry filter was applied, and fluorescence emission images were collected for monitoring purposes every 15 s for 60 min (100 ms exposure). Given a measured light power of 35 mW at the focal plane, this corresponds to an approximate intensity of 4.5 W/cm^2^.

### Selective Plane Illumination Microscope Apparatus

An in-house SPIM system was built, using a Vortran Versalase multiple wavelength system as the light source, consisting of 3 lasers (405 nm, 488 nm and 561 nm). A single mode optical fibre coupled the laser light to the SPIM illumination path, which is built as previously described^25^. The appropriate laser power was set using Stradus Versalase software, and a power meter (Thorlabs, PM100D) was used to verify collimated beam power, immediately prior to the light-sheet focussing using a cylindrical lens. The resulting beam was then focused onto the sample (10X 0.3NA Nikon CFI Fluor water dipping objective) to produce a light-sheet 400 µm high and approximately 2 µm thick at the beam waist. This corresponds to an approximate intensity of 0.37 kW/cm^2^ and 0.5 kW/cm^2^ for the light-sheet used at 3 mW and 4 mW, respectively. Larvae were embedded in 0.5% agar (in standard system water) inside an FEP tube (fluorinated ethylene propylene, Adtech), which is refractive-index-matched to water. The tube was then suspended between the imaging and illumination objectives, and orientated using a translation and rotation stage (Thorlabs). Imaging of the sample was through a 16X 0.8 NA Nikon CFI LWD Plan Fluor water dipping objective (N16LWD-PF). The resulting GFP and KillerRed emission signals were separated by a 490–550 nm dichroic beamsplitter (T550lpxr-UF2, Chroma Technology) and passed through 525/39 nm and 630/69 nm emission filters respectively (MF525-39, MF630-60, Thor Labs) onto two QI-Click Mono CCD cameras (Q-Imaging Inc). Brightfield images were separated using a 700–850 nm dichroic beamsplitter (T700spxr-UF2, Chroma Technology) and recorded on a Prosilica GS650 camera at 100 Hz. The entire system was controlled through a custom interface written in the Objective C language.

Light-sheet optoablation process: 3 dpf *ren:*mem-KillerRed;*kdrI:*GFP fish were embedded in 0.5% agar (in system water) and suspended in the FEP tubing, tail up, and oriented such that the light-sheet was directly incident upon the cerebellum, exiting via the heart. This is the optimal orientation for imaging renin cells. The 561 nm light-sheet was set to illuminate continuously, and scan across the entire volume of AMA renin expressing cells every 10 s for 1 hr. When used, the 488 nm laser was only pulsed during the camera exposure, to minimise the light dose to the specimen.

The Bessel beam (a power-adjustable Vortran Versalase multiwavelength system used at 561 nm) used as the activation laser was integrated into the SPIM through the imaging path of the system (Fig. 2a) by means of a 488/561 nm dichroic beamsplitter (Di01-R488/561-25 × 36, Semrock) placed behind the imaging objective. The beam of the activation laser was expanded (BE1) to fill the free aperture of a spatial light modulator (SLM, Holoeye LC-R 720 Spatial Light Modulator, reflective). The SLM was used to generate an axicon phase profile to produce a Bessel beam. The SLM surface was then re-imaged onto the back aperture of the imaging objective using a 4-f relay system (L1, L2). An additional phase gradient was generated using the SLM in order to direct the desired diffracted order along optical axis. An adjustable iris (Ph1) placed at the focus of the first relay lens (L1) blocked all other diffracted orders from reaching the imaging objective (OBJ2). The beam was aligned to the centre of the field-of-view of OBJ2, and small adjustments could be made by moving the mirror (M1) in order to target a specific region in the fish. The Bessel beam area within the imaging plane corresponded to intensities of approximately 1.5 kW/cm^2^, 3 kW/cm^2^ and 6 kW/cm^2^ for the 0.25 mW, 0.5 mW and 1 mW beams, respectively.

Bessel beam optoablation process: 3 dpf *ren:*mem-KillerRed fish were similarly embedded in 0.5% agar (in system water) and suspended in the FEP tubing, tail up, and optimally oriented as described for the light-sheet treatment. *ren*:mem-KillerRed^+^ cells were located using the 488 nm light-sheet, which is at the lower end of the KillerRed excitation spectrum. The Bessel beam was then translated to the region of interest with the brightest point closest to the DA. The cells of interest were continuously exposed to the laser beam and an image was acquired every 10 s for 10–20 min, until all the fluorescence had reduced to the background level.

### Multiphoton Imaging

Two-photon imaging was performed on a TriM Scope II 2-photon inverted microscope (LaVision BioTec, Germany). A pulsed Ti:Sapphire laser (Chameleon-Ultra II; Coherent, USA) was used to excite GFP at 930 nm. The KillerRed protein was excited at 1100 nm with an optical parametric oscillator laser (Chameleon Compact OPO, Coherent) pumped at 800 nm by a separate Ti:Sapphire laser source. Depth stacks of the AMA and surrounding vasculature were acquired using a 40x water immersion objective (NA = 1.15, MRD77410 Apo LWD; Nikon, Japan) with a step-size of 0.37 µm. The GFP and KillerRed emission signals were separated by a 590 nm short-pass dichroic filter (Chroma Technology), and collected through 525/70 nm and 620/60 nm bandpass filters (Chroma Technology) respectively onto a pair of photomultiplier detectors (H7422 GaAsP, Hamamatsu Photonics, Japan).

### Image Analysis

Image registration was performed using the Fiji StackReg plugin for rigid bodies^26^. During epifluorescence and Bessel beam illumination, a single image was taken per timepoint. During SPIM illumination, depth stacks were acquired across the KillerRed-expressing volume at each timepoint, and maximum intensity projections were calculated from these raw image stacks by our custom-written acquisition software. A region of interest was selected around the KillerRed-expressing cells, and background fluorescence was measured in an equivalently-sized dark region in which there were no KillerRed-expressing cells. The average intensity of the background was subtracted from the region of interest, the maximum value determined and all subsequent values calculated as a percentage of that maximum value.

### Curve fitting and Statistics

Curve fitting was performed using the curve fitting toolbox in MATLAB and the Statistics Toolbox (Release 2015b, The Mathworks, Inc., Natick, Massachusetts, USA). Double exponential fits were either used on every individual dataset and used to calculate the fast decay term half-life, or the average of each dataset within a single group compared. The parameters and R-squared value were calculated in each instance. Statistical analyses were performed with Graphpad Prism 5 (La Jolla, CA) and 95% confidence intervals are reported on each graph. A one-way ANOVA was performed on the fast decay curve half-life for each group, followed by a Tukey test for multiple comparisons.

### TUNEL staining

An ApopTag Red *In Situ* Apoptosis detection kit (Millipore, S7165) was used in conjunction with an anti-Dig-AP antibody. After optogenetic ablation procedures, *ren:*mem-KillerRed;*kdrl:*GFP fish were immediately fixed for 20 min in 4% PFA, embedded in optimal cutting temperature (OCT) compound, cryofrozen in dry-ice-cooled isopentane and sectioned sagitally in 15 μm sections. Slides were kept at −20 °C until required, then thawed to room temperature and the appropriate kidney-containing sections determined. Samples were rapidly re-fixed in 4% PFA (20 min, room temperature), permeabilised with 0.1% Triton-X and washed three times for 5 min in PBT (PBS, 0.1% tween20). Equilibrium buffer was added (45 min, room temperature), then 16 µl TdT enzyme and 30 µl reaction buffer (90 min, 37 °C, kit) were applied to the slide. The reaction was stopped using stop buffer (20 min, room temperature, kit), then incubated in blocking solution and an alkaline phosphatase-conjugated Anti-Digoxigenin (anti-Dig) (11093274910, Roche) antibody (2 hours, room temperature) and washed four times for 15 min. Sections were mounted with ProLong Gold anti-fade mountant.

### Acridine orange live staining

3 dpf *ren:*mem-KillerRed embryos were imaged incubated for 30 min in the dark in system water containing 2 μg/ml acridine orange (Sigma), followed by three 5-min washes in conditioned medium, and imaged live immediately afterwards. The light-sheet optoablation protocol was then followed, with the volume of KillerRed+ cells illuminated with the lightsheet at 4 mW every 10 s for 1 hr. Acridine orange signal was then imaged using the 488 nm lightsheet at a power of 4 mW.

## Electronic supplementary material

Supplementary Information

Supplementary Video 1
