# Supplementary material for: Precise spatio-temporal control of rapid optogenetic cell ablation with mem-KillerRed in Zebrafish
Source: Sci Rep. 2017 Jul 11;7:5096. doi: 10.1038/s41598-017-05028-2 (PMC5506062; doi:10.1038/s41598-017-05028-2)
Supplement: Supplementary file 1 — Supplementary Information [file 41598_2017_5028_MOESM1_ESM.pdf]

Precise spatio-temporal control of rapid optogenetic cell ablation with mem-KillerRed in Zebrafish

Buckley CB[1], Carvalho MT[2], Young LK[2], Rider S[1], McFadden C[1], Berlage C[2], Verdon R[2], Taylor J[3], Girkin JM[2], Mullins JJ[1]

1. BHF/University Centre for Cardiovascular Science
   University of Edinburgh
   Queen's Medical Research Institute
   47 Little France Crescent
   Edinburgh
   EH16 4TJ
2. Biophysical Sciences Institute
   Department of Physics
   Durham University
   South Road
   Durham
   DH1 3LE
3. School of Physics and Astronomy
   University of Glasgow
   Kelvin Building
   Glasgow
   G12 8QQ

Supplementary Data

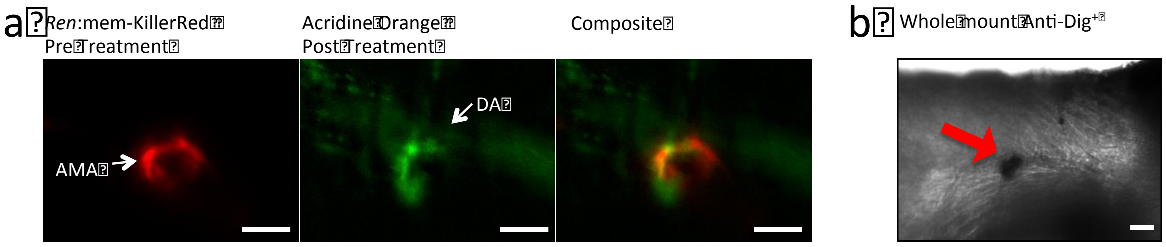

Supplementary Figure 1: Further staining to confirm cell death. (a) 3 dpf *ren*:mem-KillerRed fish were incubated in acridine orange (2 ng/ml) for 30 mins in the dark, followed by two 5 mins washes and immediately imaged live on the SPIM. Treatment with the light-sheet was performed (1 hr, 4 mW, depth-stack every 10s) and the acridine orange fluorescence signal was collected post-treatment and a composite image was created. Treated $KillerRed^+$ cells can be seen to take up acridine orange readily. (b) A whole mount *in situ* protocol was performed with an anti-Dig+ antibody, visualised using the colourant Nitro Blue Tetrazolium using the brightfield camera on the SPIM. Dig+ cells are dark spots, as indicated with the red arrow. Scale bars represent 30 μm.

Supplementary Video 1: SPIM-illuminated $KillerRed^+$ targeting in live larval zebrafish. Representative example of a 3 dpf *ren:*mem-KillerRed;k*drl*:GFP fish illuminated using the SPIM light-sheet with a power of 3 mW (561 nm laser, 1 hr, depth stack acquired every 10 s). Images were acquired axially every 1 μm across the dorsal aorta (DA) and $KillerRed^+$ AMA region, across approximately 80 μm in depth. Fish were embedded in 0.5% agar and suspended between the perpendicularly-arranged objective lenses using FEP tubing. A maximum intensity projection of the depth stacks was performed for each timepoint, and form the frames of the time-lapse movie presented at 10 images per second. The scale bar represents 30 μm.
